# A knowledge tracing approach with dual graph convolutional networks and positive/negative feature enhancement network

**DOI:** 10.1371/journal.pone.0317992

**Published:** 2025-04-09

**Authors:** Jianjun Wang, Qianjun Tang, Zongliang Zheng

**Affiliations:** 1 School of Fine Arts and Design, Leshan Normal University, Leshan, Sichuan, China; 2 School of Education Science, Leshan Normal University, Leshan, Sichuan, China; 3 School of Computer Science and Engineering, Sichuan University of Science & Engineering, Zigong, Sichuan, China; Dalian Maritime University, CHINA

## Abstract

Knowledge tracing models predict students’ mastery of specific knowledge points by analyzing their historical learning performance. However, existing methods struggle with handling a large number of skills, data sparsity, learning differences, and complex skill correlations. To address these issues, we propose a knowledge tracing method based on dual graph convolutional networks and positive/negative feature enhancement. We construct dual graph structures with students and skills as nodes, respectively. The dual graph convolutional networks independently process the student and skill graphs, effectively resolving data sparsity and skill correlation challenges. By integrating positive/negative feature enhancement and spectral embedding clustering optimization modules, the model efficiently combines student and skill features, overcoming variations in learning performance. Experimental results on public datasets demonstrate that our proposed method outperforms existing approaches, showcasing significant advantages in handling complex learning data. This method provides new directions for educational data mining and personalized learning through innovative graph learning models and feature enhancement techniques.

## Introduction

Knowledge Tracing (KT) [1–4] is a key research area in educational data mining and learning analytics. The development of KT techniques has gone through several stages [5–7], with significant research advancements from the early Bayesian Knowledge Tracing (BKT) to the more recent Deep Knowledge Tracing (DKT) [8]. However, BKT has some limitations, such as assuming the independence of knowledge points and insufficiently considering student-specific characteristics. To address these limitations, researchers have recently proposed DKT. DKT can capture more complex learning patterns and dynamic changes in knowledge states. Although DKT performs well in many respects, it also faces challenges, such as sensitivity to sequence length and limited model interpretability. However, existing methods still face challenges in handling large-scale skill sets, data sparsity, heterogeneity in learning performance, and complex inter-skill associations.

Knowledge Tracing (KT) faces several critical challenges in modern educational settings. One primary challenge is data sparsity, where students typically attempt only a small fraction of available exercises (often below 1% interaction density), making it difficult to accurately model knowledge states. Additionally, knowledge concepts are inherently interconnected through complex prerequisite and hierarchical relationships, yet traditional KT models often fail to capture these dependencies effectively. The heterogeneous nature of student learning, where individuals demonstrate varying patterns and rates of knowledge acquisition, further complicates the modeling process. Moreover, modern educational platforms must handle thousands of skills and millions of student interactions, necessitating efficient large-scale data processing while maintaining prediction accuracy. These challenges significantly impact the effectiveness of personalized learning systems and necessitate more sophisticated approaches to knowledge tracing.

This study aims to address these challenges by proposing a novel knowledge tracing prediction method based on dual graph convolutional networks and a positive/negative feature enhancement network. In recent years, Graph Neural Networks (GNNs) have excelled in processing graph-structured data, especially Graph Convolutional Networks (GCNs) [9,10]. GCNs not only excel in performance, but their intrinsic interpretability also allows researchers and engineers to better understand and optimize models. The application of GCN to KT [11,12] not only captures students’ knowledge mastery status more accurately but also flexibly handles complex learning relationships and multi-source data, significantly improving the performance and applicability of KT models. The rise of GCNs has provided a new approach, enabling us to further enhance the effectiveness of KT by modeling the relationships among students’ knowledge points. Therefore, the research motivation of this paper is to explore the application of GCNs to KT, with the expectation of improving the prediction of students’ knowledge status by capturing the complex relationships between knowledge points.

This study aims to address the challenges faced by existing KT models in dealing with large-scale skill sets, data sparsity, heterogeneity in learning performance, and complex inter-skill associations. To this end, we propose a knowledge tracing prediction method based on dual graph convolutional networks and positive /negative feature enhancement network. To begin with, to address the diversity of student behaviors and the complexity of skill relationships, we design a novel dual graph convolutional network (dual-GCN) structure that simultaneously constructs graph networks from both student and skill dimensions. Additionally, to more accurately portray the complex inter-skill correlations and enhance the predictive ability of the model, we innovatively utilize both the correct and incorrect response data of students to achieve the fusion enhancement of positive and negative features. This approach effectively improves the model’s comprehensive understanding of the learning process.

The contributions of this paper include the following:

1. We propose a knowledge tracing model based on the dual graph convolutional network and positive/negative feature enhancement network for predicting students’ knowledge mastery. Its superior performance on multiple educational datasets is experimentally verified, demonstrating improved prediction accuracy and robustness to support personalized teaching and resource optimization.
2. We propose a dual graph convolutional network that simultaneously constructs graph networks from both student and skill dimensions, enhancing the correlation between similar nodes through graph convolution operations. This structure effectively alleviates the data sparsity problem, improves the processing capability for large-scale skill sets, and better accommodates differences in student learning.
3. We propose a positive/negative feature enhancement network to bootstrap data from both positive and negative response perspectives. This network innovatively combines students’ correct and incorrect response data to comprehensively capture their knowledge status.

## Related work

### Knowledge tracing based on traditional learning

Traditional knowledge tracing methods rely on statistical and probabilistic models, typically including Bayesian Knowledge Tracing (BKT) [1,13], mixed effects models, Learning Factor Analysis (LFA) [14], and Dynamic Bayesian Networks (DBN) [15]. Bayesian models utilize Bayes’ theorem combined with prior knowledge and observational data to make inferences and predictions. The traditional BKT model assumes that all students have the same parameters in the learning of knowledge points, ignoring individual differences among students. In fact, the learning ability and learning rate of different students may vary greatly. Compared with BKT, PFA [16] proposed a new KT method that takes into account the effect of topic difficulty and the correlation between knowledge points, and has good interpretability and extensibility. Another example, Pardos et al. [17] paper improves the KT model by introducing Bayesian networks and personalization parameters, enhancing its application in education. However, these approaches have limitations in dealing with complex associations among skills and dynamic learning environments.

### Knowledge tracing based on deep learning

In order to overcome the shortcomings of traditional KT methods, more and more researchers have explored the development of KT models based on deep learning from different perspectives in recent years. KT methods based on deep learning [18,19] take more factors into account, such as the relationship between knowledge points and the consistency of the learning process, by introducing techniques such as the attention mechanism [20–22] and graph neural networks, so as to obtain more accurate student modeling and performance prediction. They embody some of the recent advances and ideas in knowledge tracing research.

The most typical deep knowledge tracing model applies a recurrent neural network (RNN) to capture the temporal dynamics of a series of interactions between students’ questions and answers. For example, DKT-DSC [23] demonstrated how deep learning techniques and dynamic classification methods can be used to improve knowledge tracing models to more accurately predict student performance and provide strong support for personalized education. DKVMN [24] proposed a knowledge tracing model based on a memory-enhanced neural network to store and retrieve knowledge states through a key-value memory mechanism. SAKT [20] applied the self-attention mechanism to KT, capturing key information in students’ answer history through attention weights. These deep models have superior performance but neglect explicit modeling of learning curve theory. Similarly, some researchers have explored feature engineering approaches. Xu et al. [25] introduced feature crosses to capture interactions between educational elements (like student-problem pairs), providing more comprehensive modeling of student learning patterns. CAKT [26] combines learning curve theory with deep learning to enhance the performance of KT models and proposes a new approach with practical applications.

### Graph-based knowledge tracing

Inspired by the representational capabilities of graph learning techniques, such as Graph Neural Networks (GNNs) [27–29], KT models based on deep learning have begun to leverage graph learning techniques to fully exploit the rich structural information in graphs and flexibly model the relationships between problems and skills [30,31]. GNNs represent questions or concepts as nodes and their relationships as edges, demonstrating advantages in modeling student learning paths and knowledge mastery [32].

Early graph-based models, such as GIKT [33] and JKT [34], demonstrated how GNNs could effectively utilize knowledge structure information [35] and model relationships between questions and knowledge points. The CMKT [36] model advanced this approach by using educational concept graphs, enhancing learner modeling and addressing data sparsity issues.

Recent innovations have focused on several key directions: (1) Interaction patterns and contrastive learning, exemplified by Bi-CLKT [37], which uses dual graph structures to simultaneously represent knowledge concepts and student learning patterns. (2) Attention mechanisms introduced through knowledge structure-aware graph attention networks [38], better capturing hierarchical relationships between concepts. (3) Heterogeneous graph networks, such as Sun et al. [39]’s tri-view contrastive learning approach based on weighted heterogeneous graphs for knowledge tracing in personalized e-learning systems, which improved tracking accuracy and robustness by integrating multi-view information of students and knowledge points through contrastive learning strategies.

The field has also made significant progress in incorporating educational theories and prior knowledge into graph-based models. Recent research has focused on combining exercise and prior knowledge differences, while dynamic cognitive diagnosis methods enhance deep knowledge tracing through educational priors. These developments demonstrate the field’s evolution toward more sophisticated and practical models that strike a balance between data-driven approaches and established educational theories.

## The proposed method

**Fig 1:**
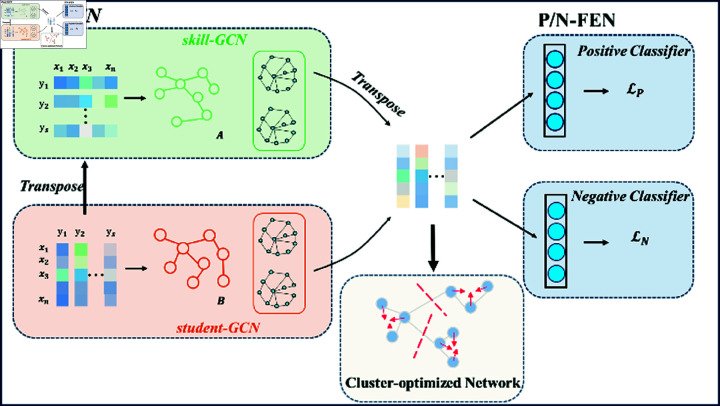
The proposed model, which is divided into three parts. The dual Graph convolution network (Dual-GCN) module, Clustering-Optimized module, and Positive/Negative Features Enhancement Network (P/N-FEN) module. A and B represent adjacency matrices, respectively.

It is a challenging task to extract effective information from the data related to KT. In order to cope with the many difficulties in KT, as shown in Fig 1, based on combining the skill information and student response information, this study proposes a knowledge tracing method based on a dual graph convolutional neural network. The proposed model is divided into three parts, Dual-GCN module, Clustering-optimized module, and P/N-FEN module. A and B represent the adjacency matrices constructed based on the dual-GCN for the input data S, respectively. Skill-GCN input features are obtained by transposing the features of student-GCN input. The transposed features generated by skill-GCN are weighted and fused with features generated by student-GCN to obtain embedded features. After that, the P/N-FEN combined with spectral embedding clustering optimization is further applied to guide the model to learn features more efficiently.

The method takes advantage of the graph structure to fully explore the correlation between students and skills, while combining students’ correct response and incorrect response data to improve the predictive ability and interpretability of the model, and thus provide personalized learning support.

### Preprocessing

#### Feature processing.

Based on the Assistments dataset, to process student skills and response data, we designed and generated three matrices (Skill Matrix: S, Response Matrix: R, and Error Response Matrix: E) to provide input and labels for subsequent knowledge tracing models. The specific steps are as follows:

1. Skill Matrix S: $S=\left[S_{1},S_{2}\ldots S_{n}\right]$, where $S_{i}$ represents a student sample, *%* . Here, *n* is the number of student samples, and *s* is the number of skills. For each sample $X_{i}$ , traverse its skill sequence. Construct a skill matrix *S* , where each row represents a sample, and each column represents a specific skill $S_{j}$ . If student *i* has used skill *j* p times, then $S_{ij}$ = p. The skill matrix S can accurately reflect each student’s mastery and application of various skills.
2. Extracting Response Matrix R: Construct a response matrix R with the same structure as the skill matrix S. For each sample $S_{i}$ , traverse its response sequence. If the corresponding response value is correct (1), set the value in the corresponding position of matrix R to 1.
3. Extracting Error Response Matrix E: Construct an error response matrix E with the same structure as the skill matrix S. For each sample $S_{i}$ , traverse its response sequence. If the corresponding response value is incorrect (0), set the value in the corresponding position of matrix E to 1.

#### Graph structure processing.

Adjacency matrices are used to represent the structure of graphs. In this study, we will construct two adjacency matrices: one is an adjacency matrix A built based on the relationships between skills, and the other is an adjacency matrix B based on student feature similarity, constructed using the K-Nearest Neighbors (KNN) method on the skill matrix S. Through these two adjacency matrices, we can more comprehensively demonstrate the interrelationships between different skills in the learning process and the similarities between students. The specific steps are as follows:

1. Adjacency Matrix Based on Skill Similarity

First, create an adjacency matrix A of size (s, s), with all initial values set to zero. If two skills appear simultaneously in the same student’s skill sequence, set the corresponding positions $A_{ij}$ and $A_{ji}$ in the adjacency matrix *A* to 1, indicating that there is a connection between these two skills. To further enhance the expressiveness of the adjacency matrix, we count the co-occurrence frequency of skills, calculate weights based on the co-occurrence frequency, and record them in the adjacency matrix *A*.


$$\begin{array}{ccc} A_{ij}=A_{ji}=\{\begin{array}{c} 0,\overset{N}{\underset{k=1}{\sum }}S_{ki}S_{kj}=0\\ \overset{N}{\underset{k=1}{\sum }}S_{ki}S_{kj},\overset{N}{\underset{k=1}{\sum }}S_{ki}S_{kj}>0 \end{array} & & \end{array}$$ (1)

Here, $A_{ij}$ represents the strength of the association between skill i and skill j.

2. Adjacency Matrix Based on Student Similarity

First, create a matrix B of size (n, n), with all initial values set to zero. Use the row vectors of the skill matrix S to represent each student’s skill usage, treating each row as a student’s feature attributes. Calculate the similarity between students to find k nearest neighbors for each student. Based on the calculated similarities, identify the k most similar students for each student. In the adjacency matrix B, set the corresponding positions $B_{ij}$ and $B_{ji}$ to 1, indicating that there is a connection between student i and student j.


$$\begin{array}{ccc} B_{ij}=B_{ji}=\{\begin{array}{c} 0,x_{i}\notin F_{K}\left(x_{j}\right)\text{ and }x_{j}\notin F_{K}\left(x_{i}\right)\\ 1,x_{i}\in F_{K}\left(x_{j}\right)\text{ or }x_{j}\in F_{K}\left(x_{i}\right) \end{array} & & \end{array}$$ (2)

Here, $B_{ij}$ represents the strength of the association between student i and student j, and $F_{K}$ denotes the KNN method used to construct the topology graph.

### Proposed model

Graph Convolutional Networks (GCNs) serve as a powerful tool with significant advantages in processing graph-structured data. Particularly in the field of knowledge tracing, GCNs can effectively capture and utilize potential similarities and commonalities among students when answering questions or using skills.

As shown in Fig 1, we propose an innovative dual graph convolutional network module to capture the relationships between students and skills more comprehensively. Our dual-GCN approach simultaneously models both student-centered and skill-centered graph structures, revealing complex interactions that single-graph methods may overlook. Specifically, we construct two graph convolutional networks: skill-GCN and student-GCN.

The dual-GCN can be intuitively understood as two complementary perspectives of the learning process. In skill-GCN, skills serve as nodes with students as node features, where frequently co-mastered skills are positioned closer together in the space. When a student masters a skill, the model can utilize this spatial relationship to better predict their performance on related skills. In student-GCN, students serve as nodes with skills as node features, where students who master similar skills are placed closer together. When one student improves in certain skills, this improvement can influence the predictions for other students with similar skill sets. Through GCN encoding of these two types of nodes, we can simultaneously capture the associations between skills and the similarities between students, thereby improving overall prediction accuracy.

To reduce the impact of different node degrees on feature aggregation, we apply symmetric normalization to the adjacency matrices A and B of skill-GCN and student-GCN during the training process.


$$\begin{array}{ccc} H^{\left(l+1\right)}=\emptyset \left({\hat{D}}^{-\frac{1}{2}}Â{\hat{D}}^{-\frac{1}{2}}H^{\left(l\right)}W^{\left(l\right)}+b^{\left(l\right)}\right) & & \end{array}$$ (3)

$$\begin{array}{ccc} H^{\prime (l+1)}=\emptyset \left({\hat{D}}^{-\frac{1}{2}}\hat{B}{\hat{D}}^{-\frac{1}{2}}H^{\prime (l)}W^{\prime (l)}+b^{\prime (l)}\right) & & \end{array}$$ (4)

Where $H^{\left(0\right)}=S^{T}$, $H^{\prime }(0)=S$, *S* and $S^{\prime }$ represent the features generated by the dual-GCN from *A* and *B* , respectively. $\hat{A}=A+I$ represents the adjacency matrix with self-loops, and $\hat{D}=\sum_{j}{\hat{A}}_{ij}$ denotes the degree matrix of $\hat{A}$ . The same applies to *B*. $S^{T}$ is the transpose of *S* . *W* , $W^{\prime }$ , *b* , and $b^{\prime }$ represent weights and bias terms, respectively. *%* denotes the activation function.

To obtain enriched node feature representations, we use a feature-weighted fusion method to process the features from the two GCNs, enhancing the interpretability of the model. The main reason for using weighted summation is that it balances the contributions of the two feature matrices in the final representation. By adjusting , we control the relative importance of the output features from the skill-GCN and student-GCN in the fused features.


$$\begin{array}{ccc} Z=\mu H^{T}+(1-\mu )H^{\prime } & & \end{array}$$ (5)

where $H^{T}\in n\times s$, $H^{\prime }\in n\times s$, *H* is the output of the skill-GCN, and $H^{\prime }$ is the output of the student-GCN.

In the clustering optimization network, we use spectral embedding techniques to generate low-dimensional node embeddings from the eigenvectors of the graph’s Laplacian matrix, preserving the structural information between nodes.

Spectral embedding clustering can be understood as a process of dimensionality reduction that preserves the essential relationships between nodes. Like arranging students in a classroom based on their learning patterns, this technique organizes nodes in a lower-dimensional space where similar nodes are grouped together. This grouping helps identify natural learning patterns and skill relationships that might not be immediately apparent in the original data.

This method helps us identify natural groups among students and skills, gaining deeper insights into the differences in skill mastery and learning behavior among different student groups. Spectral embedding optimization generates compact and highly distinguishable feature vectors, effectively clustering similar nodes together and improving the model’s accuracy in handling diverse student data.


Lclu=min ⁡ Y Tr ⁡  (YTLY) (6)

where $Y=\left[y_{1},y_{2},\ldots y_{n}\right]$ is the matrix composed of the embedding vectors of all nodes, and *Tr*() denotes the trace of the matrix.

To avoid the rotational symmetry problem of the soluton and ensure orthogonality among the vectors, we impose an orthogonal constraint on the embedding nodes:


$$\begin{array}{ccc} Y^{T}Y=I & & \end{array}$$ (7)

where for a graph *G* , its Laplacian matrix *L* is defined as *L* = *D* − *W* , with *D* being the degree matrix and *W* the adjacency matrix constructed based on the similarity of the embedded nodes *Z*.

To further encourage the model to fully explore potential association patterns between the two nodes, we designed a positive and negative feedback module to provide supervisory signals. Unlike traditional methods that focus solely on correct answers, our P/N-FEN innovatively leverages both correct and incorrect responses. This approach creates a more nuanced representation of student knowledge states, capturing subtle aspects of the learning process that are often overlooked.

Specifically, for the feature embeddings extracted earlier, we designed a positive feedback classifier for the response matrix *R* and a negative feedback classifier for the incorrect response matrix *E*. Through the joint application of positive and negative feedback classifiers, the model is effectively encouraged to explore more potential associations. The positive feedback classifier uses the response matrix *R* as positive labels, while the negative feedback classifier uses the incorrect response matrix *E* as negative labels.


$$\begin{array}{ccc} L_{P}=-\frac{1}{n}\overset{n}{\underset{i=1}{\sum }}\overset{s}{\underset{c=1}{\sum }}y_{c}^{i}log({\hat{y}}_{c}^{i}) & & \end{array}$$ (8)

$$\begin{array}{ccc} L_{N}=-\frac{1}{n}\overset{n}{\underset{i=1}{\sum }}\overset{s}{\underset{c=1}{\sum }}(1-e_{c}^{i})log({\hat{e}}_{c}^{i}) & & \end{array}$$ (9)

where *s* is the number of skills. In the positive feedback classifier, $y_{c}^{i}$ is the label for the *i* − *th* sample in the *c*–*th* skill category, and ${\hat{y}}_{c}^{i}$ is the probability predicted by the model that the *i*–*th* sample belongs to the *c*–*th* skill label. In the negative feedback classifier, $e_{c}^{i}$ is the label for the *i*–*th* sample in the *c*–*th* skill category, and ${\hat{e}}_{c}^{i}$ is the probability predicted by the model that the *i*–*th* sample belongs to the *c*–*th* skill label.

### Model training

The proposed model framework mainly consists of three modules: the dual-GCN (including skill-GCN and student-GCN), the cluster-optimized module, and the P/N-FEN module. Therefore, the total loss function *L* of the model is:


$$\begin{array}{ccc} L={\lambda L}_{clu}+\varepsilon L_{P}+\rho L_{N} & & \end{array}$$ (10)

where *λ* ,*ε* and *ρ* are hyperparameters. $L_{clu}$ is the clustering optimization loss function, $L_{P}$ is the positive feedback classifier loss function, and $L_{N}$ is the negative feedback classifier loss function.

## Experiments

### Dataset

To validate the proposed model, we conducted experiments on three public datasets [40–42] (Assistments 2009, Assistments 2012, Assistments 2017).

We processed the raw data by first reading the CSV files and removing samples containing NaN values. Then, we reordered the student IDs based on the ‘studentID’ column and reordered the skills or mapped the skill names to new skill IDs based on the ‘skill’ column. Finally, we grouped the data by student ID. As shown in Table 1, the number of student samples and the number of skill types are displayed.

**Table 1:** Overview of datasets.

	#students	#skills
Assistments 2009	4163	123
Assistments 2012	28834	198
Assistments 2017	1709	102

### Experimental setup

In this experiment, we used three different educational datasets: Assistments 2009, Assistments 2012, and Assistments 2017. These datasets represent student learning behaviors and knowledge mastery in different years, providing high representativeness and diversity. To scientifically evaluate the model’s performance, we split each dataset into 80% training and 20% testing data to ensure consistency in the distribution of the training and testing data.

To ensure the reliability and reproducibility of the experimental results, we ran each experiment five times independently and reported the average performance metrics.

### Performance comparison with different methods

To verify the effectiveness of the proposed model, we compared it with BKT [1], IRT [43], DKT [8], DKT-DSC [23], SKVMN [44], DTransformer [45], and KT-Bi-GRU [46].

As shown in Table 2, our model demonstrated excellent performance across all three datasets. Particularly on the largest dataset, Assistments 2012 (28,834 students, 198 skills), the model achieved significant advantages in both AUC (0.733) and RMSE (0.354), indicating its effective utilization of large-scale data for learning. On the smaller Assistments 2017 dataset (1,709 students, 102 skills), the model achieved the highest AUC (0.790) but showed relatively higher RMSE (0.450), suggesting strong predictive ranking ability but room for improvement in absolute value prediction on smaller datasets.

**Table 2:** Performance comparison with proposed methods on dierent datasets.

	Assistments 2009		Assistments 2012		Assistments 2017	
Metric	AUC	RMSE	AUC	RMSE	AUC	RMSE
BKT	0.692	0.418	0.677	0.392	0.773	0.450
IRT	0.753	0.462	0.712	0.400	0.683	0.433
DKT	0.728	0.422	0.673	0.385	0.620	0.319
SKVMN	0.667	0.418	0.672	0.390	0.572	**0.298**
DKT-DSC	0.725	0.421	0.694	0.387	0.781	0.452
DTransformer	0.812	0.417	0.722	0.374	0.750	0.437
KT-Bi-GRU	**0.818**	0.419	0.685	0.367	0.735	0.483
OURS	0.732	**0.415**	**0.733**	**0.354**	**0.790**	0.450

Analyzing the model structure, our proposed dual graph convolutional model captures skill relationships through skill-GCN and models student similarities through student-GCN, with this multidimensional feature extraction method showing good adaptability across datasets of different scales. Compared to traditional methods (such as BKT and IRT), our model can handle more complex nonlinear relationships; compared to other deep learning methods (such as DKT and SKVMN), our approach is more comprehensive in modeling student and skill relationships. The model also incorporates spectral embedding techniques for clustering optimization and employs positive and negative feedback classifiers, with these innovative designs collectively enhancing prediction accuracy.

Compared to recent advanced methods (such as DTransformer and KT-Bi-GRU), our model based on dual-GCN and P/N-FEN has demonstrated strong competitiveness. The model performs optimally with sufficient data (as in Assistments 2012) while maintaining stable performance with less data (as in Assistments 2017). Particularly in terms of AUC metrics, the model maintains high performance levels (0.732–0.790) across datasets of different scales, fully reflecting its adaptability and stability in complex learning environments. Although other models may perform better in specific cases (such as RMSE on Assistments 2017), our model maintains overall competitiveness and shows clear advantages on large-scale datasets.

### Ablation study of loss function

To verify the effectiveness of the extraction modules and loss functions in our proposed model, we conducted ablation experiments on three Assistments datasets of different scales. Table 3 shows the impact of different loss function combinations on the model’s performance.

**Table 3:** Ablation study of loss function.

	Assistments 2009		Assistments 2012		Assistments 2017	
Metric	AUC	RMSE	AUC	RMSE	AUC	RMSE
$L_{P}$	0.570	0.535	0.643	0.463	0.688	0.570
$L_{clu}+L_{P}$	0.685	0.541	0.710	0.411	0.722	0.559
$L_{P}+L_{N}$	0.721	0.417	0.687	0.386	0.777	0.462
$L_{clu}+L_{P}+L_{N}$	**0.732**	**0.415**	**0.733**	**0.354**	**0.790**	**0.450**

The experimental results indicate that using the positive feedback classifier loss $L_{P}$ alone yields the worst performance, suggesting that considering only the students’ correct responses is insufficient. The inclusion of the clustering loss $L_{clu}$ significantly improves the model’s performance, particularly in the AUC metric. This demonstrates the importance of spectral embedding techniques in capturing the structural relationships between students and skills. The combination of the positive feedback $L_{P}$ and negative feedback $L_{N}$ classifier losses further enhances the model’s performance, especially in the RMSE metric. This indicates that considering both the students’ correct and incorrect responses provides a more comprehensive learning signal, helping the model to predict students’ knowledge states more accurately. Finally, the complete model achieves the best or nearly best performance across all datasets. This confirms the effectiveness of our proposed multi-objective learning framework, which effectively leverages clustering information, positive feedback, and negative feedback signals, thus performing well on datasets of varying scales and characteristics.

These results not only validate the necessity of each component in our proposed model but also showcase the synergistic effects between them. By combining GCN, spectral embedding clustering, and dual feedback classifiers, our model can more comprehensively capture student-skill relationships, thereby achieving superior performance in KT tasks.

### Parameter analysis

#### Sensitivity analysis of learning rate.

To comprehensively evaluate the performance characteristics and stability of our proposed model based on dual-GCN and P/N-FEN, we conducted a detailed analysis of key hyperparameters. This section focuses on two core hyperparameters: the learning rate and the weighting of the P/N-FEN’s loss function.

**Fig 2:**
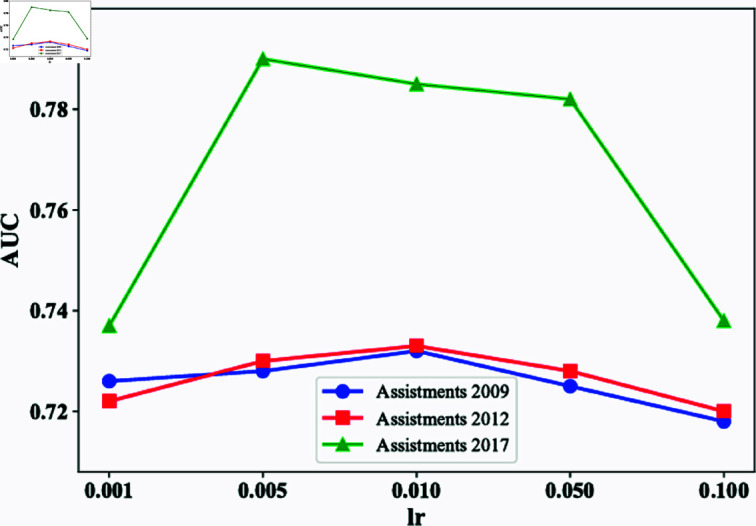
The influence of the value lr on AUC on three datasets.

The learning rate is one of the key hyperparameters that affect the model’s convergence speed and final performance. We systematically experimented with values in the range [0.001, 0.005, 0.01, 0.05, 0.1], as shown in Fig 2. We observed that there is a balance point between the learning rate and model performance: a lower learning rate may lead to a slower convergence rate, while a higher learning rate may cause instability in the training process and even degrade model performance.

#### Sensitivity analysis of the weights in the P/N enhancement loss function.

**Fig 3:**
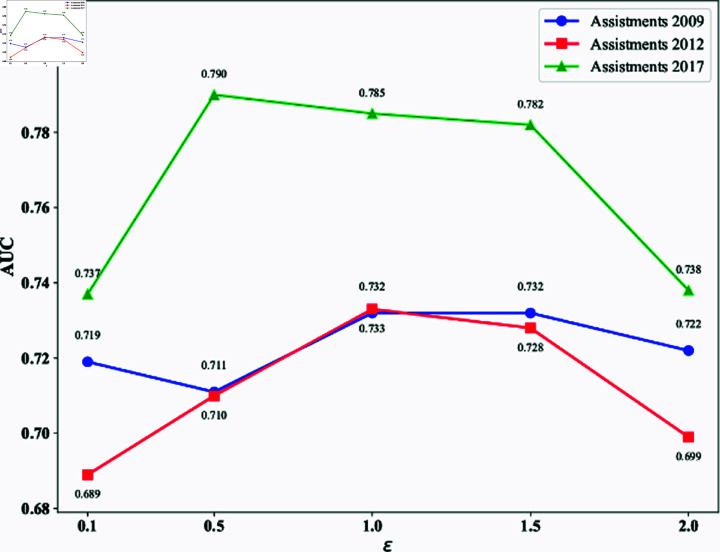
Impact of hyperparameter $\frac{\varepsilon }{\rho }$ on model performance. (a) shows the impact of $\frac{\varepsilon }{\rho }$ on model’s AUC performance, (b) shows the impact of $\frac{\varepsilon }{\rho }$ on model’s RMSE performance.

In the enhanced positive-negative loss function, weight parameters *ε* and *ρ* are crucial for balancing the model’s attention between correct and incorrect responses. To thoroughly investigate the impact of these two hyperparameters on model performance, we fixed the weight of *a* = 0 and searched for the most suitable parameter combination based on $\frac{\varepsilon }{\rho }$ . We systematically conducted experiments within the range of $\frac{\varepsilon }{\rho }=\left\{0.1,0.5,1.0,1.5,2.0\right\}$, with the experimental setup and results shown in Fig 3.

In this experiment, hyperparameters *ε* and control the weights of positive feedback loss function and negative feedback loss function in the total loss respectively, aiming to balance the model’s attention to correct and incorrect responses. Results show that different $\frac{\varepsilon }{\rho }$ ratios significantly affect AUC and RMSE across datasets. For Assistments 2009 and 2012 datasets, when $\frac{\varepsilon }{\rho }=1.0$ , AUC reached 0.732 and 0.733 respectively, with lowest RMSE at 0.415 and 0.354, showing the best performance. For the Assistments 2017 dataset, AUC peaked at 0.790 with RMSE of 0.445 when $\frac{\varepsilon }{\rho }=0.5$, indicating that higher weight on negative feedback is more suitable for this dataset. Most datasets achieved optimal performance with moderate $\frac{\varepsilon }{\rho }$ ratios (between 0.5 and 1.5), while extreme values of $\frac{\varepsilon }{\rho }$ generally led to degraded model performance. This suggests the importance of maintaining a balanced attention between correct and incorrect responses. Therefore, properly adjusting the $\frac{\varepsilon }{\rho }$ ratio is crucial for achieving optimal model performance across different datasets.

#### Robustness test.

**Fig 4:**
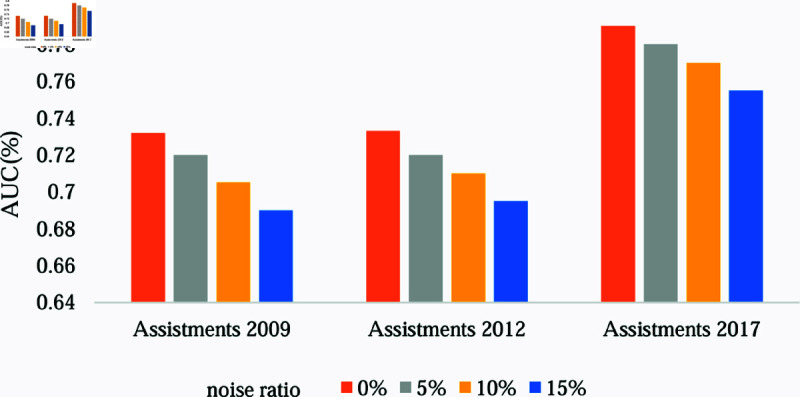
The impact of label noise on model performance.

To validate the robustness of the model under different data distributions and noise conditions, we introduced varying levels of label noise (i.e., random perturbations of correct and incorrect responses) across three datasets: Assistments 2009, Assistments 2012, and Assistments 2017. Specifically, we randomly altered 5%, 10%, and 15% of student response records in the test set. The results are shown in Fig 4.

As shown in Fig 4, the AUC values for all datasets exhibit a declining trend as the proportion of label noise increases. However, even with 15% noise, the model’s AUC remains above 0.690 across all three datasets. This demonstrates the strong robustness of our model, which can effectively resist noise interference in the data and maintain high predictive performance.

## Conclusion

With the popularization of online learning, research on knowledge tracing has gained increasing attention. Existing methods have limitations in addressing issues such as limited skill quantity, insufficient information, diversity in learning performance, and complex associations between skills. To address these problems, we propose a knowledge tracing prediction method based on dual graph convolutional networks and positive-negative feature enhancement network. We construct two graph structures with students and skills as nodes respectively. By building relationship graphs between students and skills, the model can more accurately capture and predict students’ learning behaviors and knowledge mastery levels. Meanwhile, we utilize positive and negative labels to train the model. Experimental results show that compared to existing methods, our approach demonstrates superiority and potential in handling complex learning data. However, the methods presented in this paper still have certain limitations. For example, the model primarily relies on students’ skill usage frequency and response data, and has not fully considered students’ personalized learning paths and background information. Future research can explore the introduction of more diverse features and the optimization of the graph convolution network structure to further enhance the model’s performance and adaptability.
